# Serum carbon and nitrogen stable isotopes as potential biomarkers of dietary intake and their relation with incident type 2 diabetes: the EPIC-Norfolk study^1^,^2^,^3^

**DOI:** 10.3945/ajcn.113.068577

**Published:** 2014-07-02

**Authors:** Pinal S Patel, Andrew JM Cooper, Tamsin C O'Connell, Gunter GC Kuhnle, Catherine K Kneale, Angela M Mulligan, Robert N Luben, Soren Brage, Kay-Tee Khaw, Nicholas J Wareham, Nita G Forouhi

**Affiliations:** 1 From the MRC Epidemiology Unit, University of Cambridge School of Clinical Medicine, Institute of Metabolic Science, Cambridge Biomedical Campus, Cambridge, United Kingdom (PSP, AJMC, SB, NJW, and NGF); the Department of Archaeology & Anthropology, University of Cambridge, Downing Street, Cambridge, United Kingdom (TCO); the McDonald Institute for Archaeological Research, University of Cambridge, Downing Street, Cambridge, United Kingdom (TCO and CKK); the University of Reading, Reading, United Kingdom (GGCK); and the Department of Public Health and Primary Care, University of Cambridge, Cambridge, United Kingdom (AMM, RNL, K-TK, and GGCK).

## Abstract

**Background:** Stable-isotope ratios of carbon (^13^C/^12^C, expressed as δ^13^C) and nitrogen (^15^N/^14^N, or δ^15^N) have been proposed as potential nutritional biomarkers to distinguish between meat, fish, and plant-based foods.

**Objective:** The objective was to investigate dietary correlates of δ^13^C and δ^15^N and examine the association of these biomarkers with incident type 2 diabetes in a prospective study.

**Design:** Serum δ^13^C and δ^15^N (‰) were measured by using isotope ratio mass spectrometry in a case-cohort study (*n* = 476 diabetes cases; *n* = 718 subcohort) nested within the European Prospective Investigation into Cancer and Nutrition (EPIC)–Norfolk population-based cohort. We examined dietary (food-frequency questionnaire) correlates of δ^13^C and δ^15^N in the subcohort. HRs and 95% CIs were estimated by using Prentice-weighted Cox regression.

**Results:** Mean (±SD) δ^13^C and δ^15^N were −22.8 ± 0.4‰ and 10.2 ± 0.4‰, respectively, and δ^13^C (*r* = 0.22) and δ^15^N (*r* = 0.20) were positively correlated (*P* < 0.001) with fish protein intake. Animal protein was not correlated with δ^13^C but was significantly correlated with δ^15^N (dairy protein: *r* = 0.11; meat protein: *r* = 0.09; terrestrial animal protein: *r* = 0.12, *P* ≤ 0.013). δ^13^C was inversely associated with diabetes in adjusted analyses (HR per tertile: 0.74; 95% CI: 0.65, 0.83; *P*-trend < 0.001], whereas δ^15^N was positively associated (HR: 1.23; 95% CI: 1.09, 1.38; *P*-trend = 0.001).

**Conclusions:** The isotope ratios δ^13^C and δ^15^N may both serve as potential biomarkers of fish protein intake, whereas only δ^15^N may reflect broader animal-source protein intake in a European population. The inverse association of δ^13^C but a positive association of δ^15^N with incident diabetes should be interpreted in the light of knowledge of dietary intake and may assist in identifying dietary components that are associated with health risks and benefits.

## INTRODUCTION

With the growing recognition of the importance of diet in the prevention of chronic disease (1), interest in identifying nutritional biomarkers that offer objective assessment of dietary intake to overcome limitations of imprecision, bias, and measurement error posed by the self-report of diet has increased. As a proof of principle, for incident type 2 diabetes (T2D)^4^, we previously showed a stronger inverse association with greater plasma vitamin C concentration as a marker of higher fruit and vegetable intake compared with dietary assessment by using a food-frequency questionnaire (FFQ) (2). Many other foods have been implicated in diabetes etiology, such as meat intake (3–6), fish intake (7–11), and the intake of sweetened beverages (12, 13), and the identification of biomarkers of intake of these foods has the potential to advance the field of nutritional epidemiology.

Carbon and nitrogen stable-isotope ratios (^13^C/^12^C ratio, expressed as δ^13^C, and ^15^N/^14^N ratio, expressed as δ^15^N) were recently proposed as potential nutritional biomarkers for distinguishing between meat, fish, and plant-based foods, which are isotopically distinct. The technique has a long history in archeology and ecology as a method of dietary analysis (14, 15), but few epidemiologic studies in human populations have been conducted. The most frequent application of these biomarkers to date has been described for the assessment of dietary animal protein intake, which showed that individuals consuming animal protein have higher hair δ^15^N than do those consuming vegetarian or vegan diets. For example, in the German Verbundstudie Ernährungserhebung und Risikofaktoren-Analytik study (*n* = 121 adults), hair δ^13^C and δ^15^N ratios were marginally lower among vegetarians than omnivores (16), and, in a small UK study (*n* = 28), individuals consuming diets characterized by high quantities of fish/animal protein (ie, omnivores) were more likely to have higher δ^15^N values (17). In a short-term feeding study, we showed that urinary and fecal δ^13^C and δ^15^N for individuals consuming fish and meat diets were elevated relative to those of individuals consuming a vegetarian diet, which raises the potential for their use as nutritional biomarkers of animal-derived protein intake (18). In a sample of native Alaskan Eskimos, O'Brien et al showed that δ^15^N values are strongly correlated with dietary and erythrocyte omega-3 (n−3) fatty acids (19).

The ability of the δ^13^C values of foods to distinguish between those derived from C_4_ plants (a photosynthetically distinct group of plants ie corn/maize, cane) and those from other foods derived from the more abundant C_3_ plants (eg, beets, fruit and vegetables, rice, wheat, nuts, and seeds) (20) has enabled serum δ^13^C values to be used to distinguish between individuals with a high compared with a low intake of sugar-sweetened beverages (SSBs) containing C_4_ sugars, such as high-fructose corn syrup (21). More recently, in Yup'ik Alaskan Eskimos, both erythrocyte δ^13^C and δ^15^N values predicted sweetener intake (22).

To date, the distribution of carbon and nitrogen stable isotopes in European populations has been limited to small studies of human hair (16, 17, 23, 24), whereas their potential use as nutritional biomarkers in association with disease etiology remains unexplored. The aim of this study was to 1) describe the distribution and correlates of serum δ^13^C and δ^15^N in a UK population to determine their utility as biomarkers of dietary intake, and 2) to assess the prospective association between δ^13^C and δ^15^N and incident T2D.

## SUBJECTS AND METHODS

### Study participants and design

The European Prospective Investigation into Cancer (EPIC)–Norfolk study recruited 25,639 men and women, aged 40–79 y at baseline (1993–1997), as described previously (25). In brief, since the baseline health check, there have been 3 follow-up assessments, including a postal questionnaire at 18 mo, a repeat (second) health-check visit (1998–2000) including a questionnaire, and a further postal questionnaire (2002–2004). The current analysis used data from a case-cohort design (26, 27) with 1530 participants, in which a random subcohort was selected to be representative of the whole cohort, and all incident cases were ascertained. We excluded participants with prevalent (*n* = 29) or missing diabetes status (*n* = 81); those with prevalent cardiovascular disease or cancer (*n* = 162); those without duplicate measurements of δ^15^N or δ^13^C (*n* = 7); those with >10 lines missing from the FFQ (*n* = 13); those missing data on fish, meat, or protein intake (*n* = 26); or those with missing covariate information (*n* = 34). A case-cohort study included overlap between subcohort and incident cases by design, and the final sample included 1178 participants with 476 total incident T2D cases and 718 subcohort participants (including 16 incident T2D cases and 702 noncase participants). Informed consent was obtained from all study participants, and ethical approval was granted by the Norwich District Ethics Committee.

### Baseline measurements

Health and lifestyle information was collected by using a questionnaire, including the participants’ personal and family health information, demographic circumstances, lifestyle, social circumstances, and diet (25). A standardized health check was conducted by trained nurses. Physical activity was assessed by self-report, and a validated 4-scale index was derived (inactive, moderately inactive, moderately active, and active) by combining levels of occupational and recreational physical activity (28). Smoking status was recorded in 3 categories (current, former, and never). Nonfasting venous blood samples were collected and stored overnight in a dark container at 4–7°C and then centrifuged at 2100 × *g* for 15 min at 4°C, transported, and subsequently stored in liquid nitrogen at −196°C. For plasma vitamin C measurement, venous blood was drawn into citrate-containing bottles and kept overnight in a dark container at 4–7°C, samples were centrifuged, and plasma was stabilized by using a standardized volume of metaphosphoric acid. Plasma vitamin C concentrations were measured within 1 wk of sampling by using a fluorometric assay. Serum samples were stored for an average of 12 y at −196°C before stable-isotope analysis.

### Dietary assessment

The participants completed a validated 130-item semiquantitative FFQ for food items consumed in the preceding year (29). Nutrient intake (g/d) was calculated from the frequency and amount (medium serving size) of each reported food by using in-house software (30). Protein intake from different food sources (fish, meat, dairy products, egg, vegetables, and cereal) was calculated by using McCance and Widdowson's Composition of Foods database. For instance, for fish protein, each of the 6 fish variables (fried fish in batter, fish fingers/fish cakes, other white fish, oily fish, shellfish, and fish roe) were multiplied by their corresponding protein content (protein/per gram of fish) from food codes in UK food-composition tables and were summed to obtain total fish protein. The same method was used to derive protein from other sources. Animal protein (g/d) was derived as the sum of protein from meat, fish, dairy products, and egg intakes. Terrestrial animal protein (secondary animal protein) is the sum of protein from meat, dairy products, and egg and is nonmarine (primary animal protein). Vegetable protein (g/d) was derived in a manner similar to that for fish protein by using 26 vegetable food lines. Dairy protein was derived by summing protein from milk, cream, yogurt, cheese, and dairy desserts (g/d) by using 8 dairy food lines.

#### Definition of dietary exposures

Dietary groups defined predominantly by plant, fish, or terrestrial animal intake were derived to enable comparisons with previous studies. Consumption of a vegetarian diet was addressed as a question in the health and lifestyle questionnaire (“Do you follow any particular diets?”, of which a list of diets included “vegetarian”). Participants who reported a vegetarian diet or who reported consuming no meat and no fish in the FFQ were considered to be vegetarian. Two types of consumer groups were derived to capture variation in stable isotope values: fish consumers and terrestrial animal and animal products consumers. For the fish-consumption groups, we included only lower meat consumers (<100 g/d) and then classified participants on the basis of their levels of fish consumption (based on the number of portions of fish consumed per week, assuming a portion size of 100 g); the levels were as follows: nonconsumer, low consumer (>0 to ≤1 portion/wk); medium consumer (>1 to ≤2 portions/wk); high consumer (>2 to ≤3 portions/wk); and very high consumer (>3 portions/wk). For the terrestrial animal and animal product consumption groups, we included only lower fish consumers (<200 g/wk) and then classified participants according to their level of combined intakes of meat, dairy products, and eggs; the levels were as follows: low consumer (<250 g/d), medium consumer (250 to ≤500 g/d), high consumer (500 to ≤750 g/d), and very high consumer (>750 g), with 250-g/d increments being half of the mean consumption among low fish eaters (mean = 508 g/d; range = 0–1089 g/d). Because of homogeneity in consumption of fish and meat in this study population, the consumer groups were not mutually exclusive. In addition, consumer groups were defined to create strata that contained sufficient data that would allow for comparisons. For these reasons, the analyses including these definitions should be interpreted with caution.

#### Fatty acid analysis

To enable the examination of the association between isotopic values (δ^13^C and δ^15^N) and omega-3 fatty acids, as previously described for δ^15^N (19), we used a subset of our sample (*n* = 147) with measures of plasma phospholipid fatty acids (31) for α-linolenic acid (ALA), EPA, docosapentaenoic acid, and DHA expressed as a percentage of the total fatty acid concentrations (31).

### Stable isotopic analysis

Sample preparation was carried out at the MRC Epidemiology Unit, University of Cambridge, United Kingdom. Duplicate 10-μL aliquots of serum were drawn from thawed samples, transferred into high-purity tin capsules (3.5 × 3.75 mm), and then dried in a speed vacuum concentrator (miVAC; Genevac Ltd). Samples were prepared in duplicate batches and transported within 1 wk of preparation to the McDonald Institute for Archaeological Research, University of Cambridge. Isotopic analyses were performed by using a Costech automated elemental analyzer coupled in continuous-flow mode to an isotope ratio–monitoring mass spectrometer (Thermo Finnigan MAT253) at the Godwin Laboratory, Department of Earth Sciences, University of Cambridge. Stable isotope concentrations are measured as the ratio of the heavier isotope to the lighter isotope relative to an internationally defined scale, Vienna Pee Dee Belemnite for carbon and mean atmospheric nitrogen for nitrogen, according to standard convention (32). The natural abundance stable-isotope composition of δ^13^C and δ^15^N are reported in the delta (δ) scale in parts per thousand or “per mil” (expressed as ‰) values, where δX = [(R_sample_/R_standard_) − 1] × 1000, where R is the ratio of heavy to light isotope (for both nitrogen and carbon). The δ^13^C and δ^15^N values in this study are reported as the mean of samples analyzed in duplicate. Based on replicate analyses of international and laboratory standards, measurement errors were ≤0.2‰ for δ^13^C and δ^15^N for 92% of the study population (*n* = 1,178) and ≤0.9‰ for the remaining 8%.

### Diabetes case ascertainment

Incident cases of diabetes occurring until 31 July 2006 were ascertained by using multiple sources, including self-report of doctor-diagnosed diabetes or diabetes-specific medication, linkage to primary or secondary care registers, hospital admissions data, or mortality data. Follow-up began at the date of recruitment and ended at either 31 July 2006, date of diabetes diagnosis, or date of death (if the individual died before this date), whichever occurred first. Individuals with prevalent diabetes or for whom the diabetes status could not be confirmed were excluded from analysis.

### Statistical analysis

#### Distribution of δ^13^C and δ^15^N values and baseline characteristics

We examined the mean and SD of δ^13^C and δ^15^N values in the entire subcohort population and described the sociodemographic and dietary characteristics of participants by tertiles of both biomarkers separately by using means (±SDs), medians (IQRs), or frequencies. We created bag plots (33) to examine pictorially the combined distribution of δ^13^C and δ^15^N in the subcohort by dietary status characterized by vegetarian diet, fish-consumer status, and terrestrial animal consumer status and tested joint differences in isotope ratios across these groups by using a multivariate test of means (mvtest command).

#### Correlation between stable isotopes and dietary intake

Spearman correlation coefficients (Spearman's rho, *r*) were calculated to examine the relation between δ^13^C and δ^15^N values and dietary intake, including dietary protein intake (total and from fish, meat, dairy products, terrestrial animal, vegetables, and cereals) and sugar intake (total, fructose, glucose, galactose, lactose, and sucrose). In the subgroup with plasma phospholipid measures available (*n* = 147 in the subcohort), we also examined Spearman correlations with total omega-3 fatty acids, ALA, EPA, docosapentaenoic acid, and DHA.

#### Association between stable isotopes, dietary factors, and incident diabetes

To estimate the association between stable isotopes and types of dietary protein intake and diabetes, we used Prentice-weighted Cox proportional hazards analysis, which accounts for the case-cohort design, and with age as the underlying time variable (26, 27).

HRs and corresponding 95% CIs for diabetes comparing tertiles of δ^13^C and δ^15^N were examined for the association between stable isotopes and incident diabetes. We also examined the association of total protein and protein from fish, meat, terrestrial animal, dairy products, vegetables, and cereals with diabetes. We used the following modeling strategy to adjust for potential confounders: in model 1 we adjusted for sex, family history of diabetes (2 categories), smoking status (3 categories), educational level (4 categories), physical activity level (4 categories), and δ^13^C (for δ^15^N analyses) or δ^15^N (for δ^13^C analyses). In model 2 we additionally adjusted for dietary risk factors, including total energy intake (kcal/d), alcohol intake (g/d), and plasma vitamin C (μmol/L) (as an objective marker of fruit and vegetable intakes). Model 3 was further adjusted for BMI (continuous) and waist circumference (continuous). In model 4 we also adjusted for terrestrial animal protein intake to examine the possibility that intake may mediate the association between δ^13^C and δ^15^N and diabetes. In sensitivity analyses we also *1*) adjusted for fish protein in model 4 to test the independent association between δ^13^C, δ^15^N, meat- and terrestrial animal-protein, and diabetes and *2*) excluded individuals with δ^13^C or δ^15^N measurement >0.2‰ (ie, the cutoff used for analytic uncertainty in δ^13^C or δ^15^N measurement, *n* = 97 of 1178). All statistical analyses were performed by using STATA version 11.2 (StataCorp). Bag plots were created by using version 2.12.1 of the statistical package R (34).

## RESULTS

The mean (±SD) age of the subcohort participants was 57.7 ± 9.3 y, the mean (±SD) BMI (in kg/m^2^) was 25.9 ± 3.6, 41% were men, 13% reported being current smokers and 38% former smokers, 47% reported being physically or moderately physically active, and 37% reported having finished education after compulsory schooling (age 16 y). The mean (±SD) value for δ^13^C was −22.8 ± 0.4‰ (a negative delta value for δ^13^C means that the isotopic ratio of the sample is lower than that of the standard) and for δ^15^N was 10.2 ± 0.4‰. The Pearson correlation (*r*) value between δ^13^C and δ^15^N was *r* = 0.28 (*P* < 0.001) in the subcohort and was *r* = 0.26 (*P* < 0.001) in the cases.

### Baseline demographic and dietary characteristics by δ^13^C and δ^15^N distribution

Smoking frequency was higher across increasing tertiles of both δ^13^C and δ^15^N, and participants were older across increasing tertiles of δ^15^N; however, no other sociodemographic factors varied with isotopic values (**Tables 1** and **2**). Of the dietary factors, dietary fish protein had the strongest correlations (*r* = 0.22 and *P* < 0.001 for δ^13^C; *r* = 0.20 and *P* < 0.001 for δ^15^N), which were not materially different among incident cases only (results not shown). Whereas increasing δ^13^C values were positively correlated with fish intake and fish protein intake, no significant association of intakes of meat, meat protein, terrestrial animal, or terrestrial animal protein intake with δ^13^C was observed. With increasing δ^15^N values, there were higher intakes of fish and dairy products, higher terrestrial animal intakes, a lower frequency of a vegetarian diet, and higher intakes of animal-source protein (including meat protein, dairy protein, and terrestrial animal protein). Of the sugars, only lactose intake was higher across increasing δ^15^N, with a modest correlation (*r* = 0.12, *P* = 0.001); no variation across δ^13^C values was observed. Intakes of total breakfast cereals, fizzy drinks, or fruit juice did not differ by tertiles of δ^13^C or δ^15^N (data not shown), whereas a higher alcohol intake was observed with increasing δ^13^C. Cereal protein intake was inversely associated with both δ^13^C (*r* = −0.13, *P* < 0.001) and δ^15^N (*r* = −0.10, *P* = 0.011).

**TABLE 1:** Baseline sociodemographic and dietary characteristics by tertiles of δ^13^C values in the subcohort (*n* = 718): the EPIC-Norfolk study*^1^*

	Tertiles of δ^13^C values				Correlation analyses*^2^*	
	1	2	3	*P*-trend*^3^*	Spearman's *r*	*P* value
*n*	245	234	239	—	—	—
δ^13^C (‰)	−23.2 ± 0.2*^4^*	−22.8 ± 0.1	−22.4 ± 0.3	<0.001	—	—
Range	−23.83 to −22.97	−22.96 to −22.68	−22.67 to −21.12	—	—	—
δ^15^N (‰)	10.1 ± 0.4	10.3 ± 0.4	10.4 ± 0.4	<0.001	—	—
Range	8.57–11.29	9.16–11.36	8.03–11.67	—	—	—
Sociodemographic characteristics						
Age (y)	57.0 ± 9.7	57.7 ± 9.3	58.3 ± 9.0	0.36	0.05	0.16
Male sex [*n* (%)]	92 (37.6)	91 (38.9)	111 (46.4)	0.10	—	—
BMI (kg/m^2^)	25.7 ± 3.4	26.1 ± 3.5	25.9 ± 3.8	0.47	0.01	0.75
Waist circumference (cm)						
Men	92.9 ± 9.0	95.5 ± 9.0	94.9 ± 10.4	0.16	0.09	0.10
Women	81.0 ± 9.9	80.9 ± 9.2	80.4 ± 10.7	0.87	−0.03	0.53
Family history of diabetes [*n* (%)]	32 (13.1)	19 (8.1)	28 (11.7)	0.21	—	—
Smoking status [*n* (%)]				0.047		
Never	125 (51.0)	124 (53.0)	99 (41.4)		—	—
Former	92 (37.6)	85 (36.3)	98 (41.0)		—	—
Current	28 (11.4)	25 (10.7)	42 (17.6)		—	—
Educational level [*n* (%)]				0.33	—	—
Compulsory schooling	100 (40.8)	84 (35.9)	80 (33.5)		—	—
Up to O level	16 (6.5)	25 (10.7)	29 (12.1)		—	—
Up to A level	99 (40.4)	90 (38.5)	95 (39.8)		—	—
Up to degree level	30 (12.2)	35 (15.0)	35 (14.6)		—	—
Physical activity [*n* (%)]				0.09	—	—
Active	55 (22.5)	55 (23.5)	41 (17.2)		—	—
Moderately active	54 (22.0)	55 (23.5)	78 (32.6)		—	—
Moderately inactive	75 (30.6)	69 (29.5)	58 (24.3)		—	—
Inactive	61 (24.9)	55 (23.5)	62 (25.9)		—	—
Dietary characteristics						
Total energy intake (kcal/d)	2083 ± 604	2056 ± 645	1999 ± 560	0.30	−0.04	0.26
Fat (g/d)	79.9 ± 31.7	77.6 ± 31.7	73.4 ± 30.0	0.07	−0.08	0.03
Carbohydrate (g/d)	261.9 ± 81.2	258.7 ± 87.7	247.4 ± 75.2	0.12	−0.06	0.12
Alcohol intake (g/d)	2.4 (0.8, 7.0)*^5^*	4.6 (0.8, 10.6)	6.1 (1.3, 13.0)	<0.001	0.20	<0.001
Fruit intake (g/d)	221 (132, 338)	193 (112, 344)	218 (124, 333)	0.44	−0.02	0.54
Vegetable intake (g/d)	256 (195, 348)	241 (180, 349)	248 (186, 323)	0.35	−0.04	0.28
Fish intake (g/d)	27 (16, 39)	32 (20, 44)	39 (25, 57)	<0.001	0.22	<0.001
Meat intake (g/d)	100 (65, 136)	94 (65, 126)	99 (69, 125)	0.75	−0.008	0.83
Dairy intake (g/d)	371 (291, 508)	362 (298, 527)	399 (308, 528)	0.29	0.06	0.13
Terrestrial animal intake (g/d)*^6^*	510 (375, 626)	480 (375, 663)	510 (405, 655)	0.41	0.05	0.18
Follows a vegetarian diet [*n* (%)]	16 (6.5)	7 (3.0)	14 (5.9)	0.18	—	—
Total sugars (g/d)	126.1 (97.4, 157.2)	124.7 (95.0, 159.1)	125.6 (97.9, 154.4)	0.90	0.01	0.77
Fructose (g/d)	22.9 (16.9, 31.3)	21.7 (15.9, 30.0)	22.7 (16.8, 29.2)	0.62	−0.02	0.56
Glucose (g/d)	21.1 (15.7, 27.2)	20.3 (14.9, 27.6)	20.7 (15.2, 27.0)	0.69	−0.03	0.44
Galactose (g/d)	0.2 (0.0, 0.8)	0.2 (0.0, 0.8)	0.2 (0.0, 0.8)	0.25	0.05	0.16
Lactose (g/d)	19.9 (15.1, 26.5)	19.0 (14.8, 28.4)	20.5 (16.4, 27.6)	0.28	0.05	0.22
Sucrose (g/d)	50.4 (34.9, 71.0)	49.2 (35.4, 71.9)	52.0 (34.4, 70.3)	0.99	0.03	0.48
Total protein (g/d)	82.2 (66.1, 95.6)	81.2 (66.6, 95.8)	80.8 (67.2, 94.2)	0.95	0.007	0.86
Fish protein (g/d)	5.1 (3.3, 7.4)	5.6 (4.1, 8.2)	7.2 (4.7, 10.3)	<0.001	0.22	<0.001
Meat protein (g/d)	24.1 (14.8, 32.4)	23.2 (16.0, 31.1)	24.3 (16.6, 30.0)	0.92	0.009	0.81
Dairy protein (g/d)	16.4 (12.6, 21.0)	16.7 (11.8, 22.2)	17.2 (13.2, 21.6)	0.54	0.04	0.24
Terrestrial animal protein (g/d)*^6^*	41.8 (32.5, 53.0)	43.0 (33.3, 51.6)	43.0 (34.5, 52.2)	0.88	0.04	0.33
Vegetable protein (g/d)	5.6 (4.2, 7.8)	5.5 (4.2, 7.5)	5.3 (3.8, 7.2)	0.21	−0.05	0.14
Cereal protein (g/d)	9.2 (6.1, 13.4)	8.9 (5.7, 12.6)	7.4 (5.2, 11.0)	0.003	−0.13	<0.001

1 EPIC, European Prospective Investigation into Cancer and Nutrition; FFQ, food-frequency questionnaire; δ^13^C, carbon stable-isotope ratio (^13^C/^12^C ratio); δ^15^N, nitrogen stable-isotope ratio (^15^N/^14^N ratio).

2 Spearman's rho, *r*, between δ^13^C values (‰) and total and types of FFQ-derived dietary intake (g/d). Differences across tertiles are denoted by *P* values, which correspond to Kruskal-Wallis test.

3 *P*-trend across tertiles of δ^13^C.

4 Mean ± SD (all such values).

5 Median; IQR in parentheses (all such values).

6 Terrestrial animal protein intake is the sum of meat, dairy, and egg protein (g/d).

**TABLE 2:** Baseline sociodemographic and dietary characteristics by tertiles of δ^15^N values in the subcohort (*n* = 718): the EPIC-Norfolk study*^1^*

	Tertiles δ^15^N values				Correlation analyses*^2^*	
	1	2	3	*P*-trend*^3^*	Spearman's *r*	*P* value
*n*	248	235	235	—	—	—
δ^15^N (‰)	9.8 ± 0.3*^4^*	10.3 ± 0.10	10.7 ± 0.2	<0.001	—	—
Range	8.03 to 10.09	10.10 to 10.43	10.44 to 11.67	—	—	—
δ^13^C (‰)	−22.9 ± 0.4	−22.8 ± 0.4	−22.7 ± 0.3	<0.001	—	—
Range	−23.83 to −21.43	−23.76 to −21.12	−23.69 to −21.28	—	—	—
Sociodemographic characteristics					—	—
Age (y)	56.1 ± 9.7	58.0 ± 9.2	59.0 ± 8.8	0.002	0.14	<0.001
Male sex [*n* (%)]	111 (44.8)	92 (39.2)	91 (38.7)	0.32	—	—
BMI (kg/m^2^)	25.7 ± 3.6	26.0 ± 3.6	26.0 ± 3.5	0.58	0.06	0.09
Waist circumference (cm)						
Men	93.3 ± 9.2	95.2 ± 10.6	95.3 ± 9.0	0.24	0.07	0.26
Women	80.9 ± 10.8	80.9 ± 10.0	80.5 ± 8.7	0.93	0.04	0.37
Family history of diabetes [*n* (%)]	30 (12.1)	22 (9.4)	27 (11.5)	0.61	—	—
Smoking status [*n* (%)]				0.002	—	—
Never	111 (44.8)	128 (54.5)	109 (46.4)		—	—
Former	113 (45.6)	81 (34.5)	81 (34.5)		—	—
Current	24 (9.7)	26 (11.1)	45 (19.2)		—	—
Educational level [*n* (%)]				0.56	—	—
Compulsory schooling	84 (33.9)	93 (39.6)	87 (37.0)		—	—
Up to O level	22 (8.9)	27 (11.5)	21 (8.9)		—	—
Up to A level	106 (42.7)	81 (34.5)	97 (41.3)		—	—
Up to degree level	36 (14.5)	34 (14.5)	30 (12.8)		—	—
Physical activity [*n* (%)]				0.22	—	—
Active	57 (23.0)	51 (21.7)	43 (18.3)		—	—
Moderately active	63 (25.4)	58 (24.7)	66 (28.1)		—	—
Moderately inactive	71 (28.6)	75 (31.9)	56 (23.8)		—	—
Inactive	57 (23.0)	51 (21.7)	70 (29.8)		—	—
Dietary characteristics						
Total energy intake (kcal/d)	2059 (570)	2016 (596)	2065 (647)	0.63	−0.02	0.55
Fat (g/d)	77.4 (29.9)	74.5 (29.1)	79.1 (34.4)	0.27	−0.002	0.96
Carbohydrate (g/d)	260.6 (79.2)	253.7 (80.2)	253.6 (85.6)	0.56	−0.06	0.10
Alcohol intake (g/d)	3.3 (0.8, 9.7)*^5^*	4.1 (0.8, 9.9)	4.7 (0.8, 11.5)	0.82	0.03	0.45
Fruit intake (g/d)	213 (123, 341)	219 (133, 353)	204 (115, 330)	0.63	−0.02	0.51
Vegetable intake (g/d)	250 (182, 340)	242 (187, 338)	248 (187, 333)	0.90	−0.02	0.65
Fish intake (g/d)	27 (16, 43)	32 (22, 46)	38 (27, 51)	<0.001	0.21	<0.001
Meat intake (g/d)	94 (61, 124)	98 (65, 132)	103 (73, 136)	0.16	0.07	0.06
Dairy intake (g/d)	335 (249, 515)	367 (304, 508)	431 (312, 560)	0.004	0.13	<0.001
Terrestrial animal intake (g/d)*^6^*	464 (355, 631)	502 (382, 649)	543 (418, 670)	0.004	0.13	<0.001
Follows a vegetarian diet [*n* (%)]	21 (8.5)	11 (4.7)	5 (2.1)	0.006	—	**—**
Total sugars (g/d)	126.4 (95.6, 156.1)	125.1 (96.9, 153.6)	125.6 (99.9, 159.1)	0.70	0.003	0.94
Fructose (g/d)	22.6 (16.2, 30.0)	22.1 (16.2, 30.2)	22.6 (16.5, 29.7)	0.97	−0.007	0.85
Glucose (g/d)	21.1 (15.2, 27.2)	20.5 (15.3, 27.8)	19.9 (15.2, 26.5)	0.58	−0.04	0.34
Galactose (g/d)	0.2 (0.0, 0.8)	0.2 (0.0, 0.8)	0.2 (0.0, 0.8)	1.00	0.002	0.96
Lactose (g/d)	18.2 (13.6, 27.1)	19.9 (15.8, 26.3)	21.6 (16.5, 29.6)	0.005	0.12	0.001
Sucrose (g/d)	51.9 (34.8, 74.4)	48.7 (35.8, 66.8)	50.7 (34.9, 70.3)	0.61	−0.02	0.65
Total protein (g/d)	79.3 (66.4, 93.2)	81.8 (65.6, 95.7)	83.7 (68.5, 97.9)	0.21	0.07	0.08
Fish protein (g/d)	5.1 (3.3, 8.2)	5.7 (3.6, 8.3)	6.9 (4.7, 9.2)	<0.001	0.20	<0.001
Meat protein (g/d)	22.2 (14.0, 29.6)	23.7 (15.5, 31.9)	24.8 (16.9, 31.7)	0.051	0.09	0.013
Dairy protein (g/d)	15.9 (11.8, 21.1)	16.7 (13.0, 21.1)	18.0 (13.2, 22.0)	0.016	0.11	0.003
Terrestrial animal protein (g/d)*^6^*	41.5 (32.7, 48.9)	43.0 (31.9, 52.4)	43.9 (35.3, 54.3)	0.010	0.12	0.002
Vegetable protein (g/d)	5.5 (4.1, 8.1)	5.6 (4.0, 7.5)	5.2 (4.0, 7.2)	0.20	−0.07	0.07
Cereal protein (g/d)	8.8 (5.9, 14.0)	9.2 (5.6, 12.0)	7.5 (5.2, 11.6)	0.020	−0.10	0.011

1 EPIC, European Prospective Investigation into Cancer and Nutrition; FFQ, food-frequency questionnaire; δ^13^C, carbon stable-isotope ratio (^13^C/^12^C ratio); δ^15^N, nitrogen stable-isotope ratio (^15^N/^14^N ratio).

2 Spearman's rho, *r*, between δ^15^N values (‰) and total and types of FFQ-derived dietary intake (g/d). Differences across tertiles are denoted by *P* values, which correspond to Kruskal-Wallis test.

3 *P*-trend across tertiles of δ^15^N.

4 Mean ± SD (all such values).

5 Median; IQR in parentheses (all such values).

6 Terrestrial animal protein intake is the sum of meat, dairy, and egg protein (g/d).

**FIGURE 1.**
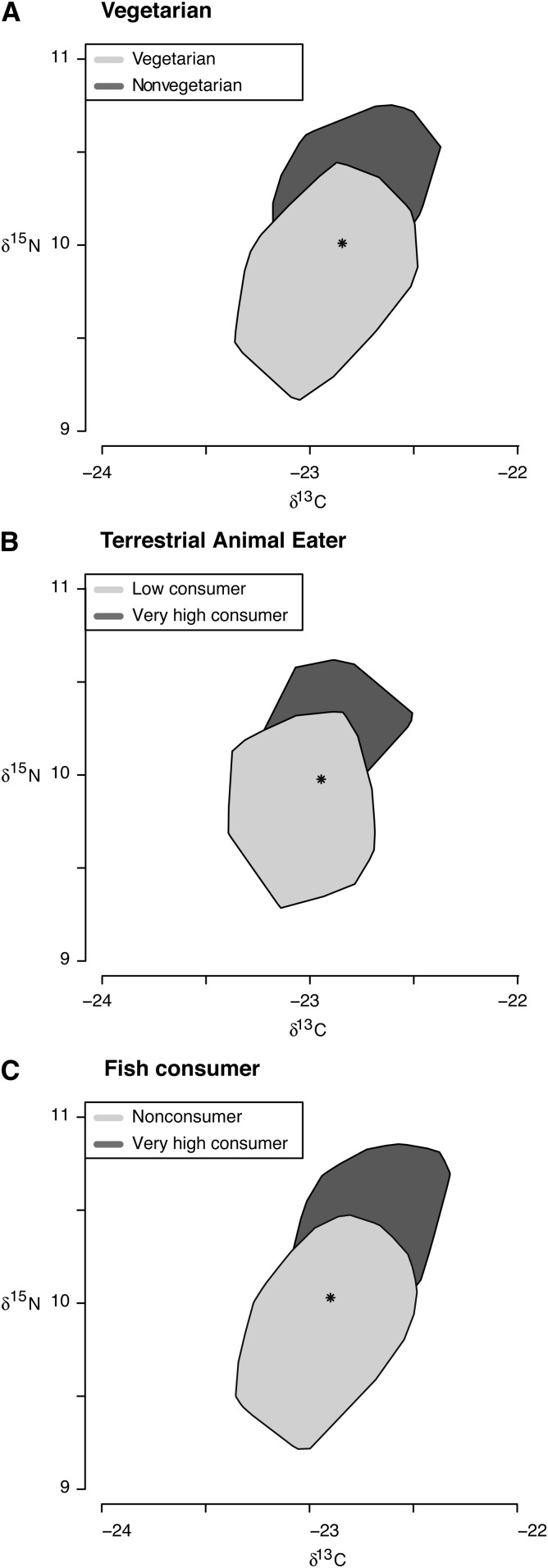
Bag plots of δ^13^C and δ^15^N (‰) distribution by vegetarian status (yes, *n* = 37; no, *n* = 681) (A), terrestrial animal product consumer (very high consumption, *n* = 32; low consumption, *n* = 28) (B), and fish consumer (very high consumption, *n* = 92; no consumption, *n* = 43) (C). Bag plots were generated to examine pictorially the combined distribution of δ^13^C and δ^15^N by dietary status characterized by vegetarian compared with nonvegetarian diet, terrestrial animal consumer status, and fish-consumer status. Joint differences in isotope ratios across consumption groups were tested by using a multivariate test of means (*P* < 0.001 for all comparisons). δ^13^C, carbon stable-isotope ratio (^13^C/^12^C ratio); δ^15^N, nitrogen stable-isotope ratio (^15^N/^14^N ratio).

Both mean δ^13^C and δ^15^N values were higher with greater fish-consumer status, whereas only δ^15^N values were higher with greater terrestrial animal and animal product consumption (*see* Supplementary Table 1 under “Supplemental data” in the online issue). The bag plots in **Figure 1** visually compare the combined variation in mean δ^13^C and δ^15^N values by dietary status, showing statistically significant differences between nonvegetarians and vegetarians (A; *P* < 0.001), low and very high terrestrial animal products consumers (B; *P* < 0.001), and non- and very high fish consumers (C; *P* < 0.001). *P* values represent significant differences across 4 categories for terrestrial animal consumption and across 5 categories for fish consumption.

### Association of stable isotopes with plasma phospholipid fatty acids

Of the plasma phospholipid fatty acids (*n* = 147 participants), positive correlations were observed for EPA with δ^13^C (*r* = 0.23, *P* < 0.01) and δ^15^N (*r* = 0.22, *P* < 0.01) and for total omega-3 fatty acids (*r* = 0.26, *P* < 0.01) and DHA (*r* = 0.30, *P* < 0.001) with δ^15^N. In addition, ALA was inversely associated with δ^13^C (*r* = −0.24, *P* < 0.01) and δ^15^N (−0.21, *P* < 0.01). Median (IQR) fatty acids relative to total fatty acids were as follows: total omega-3, 7.4% (6.4, 8.7%); ALA, 0.2% (0.2, 0.3%); EPA, 1.1% (0.7, 1.4%); and DHA, 4.8% (4.0, 5.6%).

### Association between stable isotopes, dietary factors, and the risk of diabetes

Compared with the subcohort, persons who developed diabetes were older, were more likely to be male and to have a family history of diabetes, had a higher BMI and waist circumference, were more physically inactive, were less likely to smoke, and were less likely to be educated to a degree level (*see* Supplementary Table 2 under “Supplemental data” in the online issue). The case participants reported higher intakes of meat (*P* < 0.001) but lower intakes of fruit (*P* = 0.01) and fruit juice (*P* = 0.04) and had lower plasma vitamin C concentrations (*P* < 0.001). Mean (±SD) δ^15^N values were slightly but significantly higher in cases (10.3 ± 0.4‰) than in the subcohort (10.2‰ ± 0.4; *P* = 0.006). Conversely, δ^13^C values were slightly but significantly lower (*P* = 0.009) in cases (−22.9 ± 0.4‰) than in the subcohort (−22.8 ± 0.4‰).

The δ^13^C values were inversely associated with diabetes in adjusted analyses (HR per tertile: 0.74; 95% CI: 0.65, 0.83; *P*-trend < 0.001; model 3), as shown in **Table 3**. In contrast, δ^15^N values were positively associated with diabetes (HR per tertile: 1.23; 95% CI: 1.09, 1.38; *P*-trend = 0.001, model 3). Additional adjustment for terrestrial animal protein intake did not materially change the associations observed between δ^13^C, δ^15^N, and diabetes (model 4). Fish protein intake was inversely associated with diabetes, whereas terrestrial animal and meat protein intakes were positively associated with diabetes; dairy product, vegetable, and cereal protein intakes were not associated with diabetes. In sensitivity analyses, adjustment for fish protein intake (model 4) did not materially change the associations between δ^13^C, δ^15^N, meat protein, or terrestrial animal protein and diabetes. In addition, exclusion of samples with a >0.2‰ range (*n* = 97) in δ^13^C and δ^15^N duplicate measurements did not alter our findings.

**TABLE 3:** The association between tertiles of δ^13^C and δ^15^N values, types of dietary protein intake, and type 2 diabetes: the EPIC-Norfolk Study*^1^*

	Tertiles of stable isotopes and dietary protein types*^2^*				
	1	2	3	Effect per tertile	*P-*trend
δ^13^C					
Cases/subcohort	205/245	126/234	145/239	—	
Median (‰)	−23.1	−22.8	−22.5	—	
Model 1	1	0.66 (0.53, 0.83)	0.60 (0.48, 0.75)	0.77 (0.69, 0.86)	<0.001
Model 2	1	0.64 (0.50, 0.80)	0.60 (0.48, 0.76)	0.77 (0.68, 0.86)	<0.001
Model 3	1	0.66 (0.52, 0.84)	0.55 (0.43, 0.69)	0.74 (0.65, 0.83)	<0.001
Model 4	1	0.68 (0.54, 0.87)	0.55 (0.43, 0.70)	0.75 (0.65, 0.83)	<0.001
δ^15^N					
Cases/subcohort	131/248	170/235	175/235	—	
Median (‰)	9.9	10.3	10.7	—	
Model 1	1	1.27 (1.01, 1.61)	1.23 (0.97, 1.55)	1.10 (0.98, 1.23)	0.10
Model 2	1	1.32 (1.05, 1.67)	1.22 (0.96, 1.54)	1.09 (0.98, 1.23)	0.12
Model 3	1	1.52 (1.19, 1.95)	1.55 (1.22, 1.99)	1.23 (1.09, 1.38)	0.001
Model 4	1	1.53 (1.19, 1.96)	1.54 (1.21, 1.97)	1.22 (1.09, 1.38)	0.001
Fish protein					
Cases/subcohort	179/240	140/240	157/238	—	
Median (g/d)	3.3	5.9	10.4	—	
Model 1	1	0.75 (0.59, 0.93)	0.95 (0.76, 1.18)	0.97 (0.86, 1.08)	0.56
Model 2	1	0.74 (0.59, 0.92)	1.00 (0.80, 1.25)	0.99 (0.88, 1.11)	0.85
Model 3	1	0.63 (0.50, 0.80)	0.73 (0.57, 0.92)	0.84 (0.74, 0.95)	0.006
Model 4	1	0.63 (0.50, 0.79)	0.72 (0.57, 0.92)	0.84 (0.74, 0.95)	0.005
Terrestrial animal protein *^3^*					
Cases/subcohort	133/239	152/240	191/239	—	
Median (g/d)	29.7	42.6	58.5	—	
Model 1	1	1.05 (0.82, 1.33)	1.46 (1.16, 1.83)	1.22 (1.09, 1.37)	0.001
Model 2	1	1.14 (0.89, 1.46)	1.65 (1.27, 2.13)	1.29 (1.14, 1.47)	<0.001
Model 3	1	0.87 (0.68, 1.12)	1.56 (1.19, 2.05)	1.27 (1.10, 1.46)	0.001
Model 4	1	0.87 (0.68, 1.12)	1.58 (1.20, 2.07)	1.28 (1.11, 1.47)	0.001
Meat protein					
Cases/subcohort	118/239	174/240	184/239	—	
Median (g/d)	12.5	23.7	36.2	—	
Model 1	1	1.28 (1.00, 1.63)	1.40 (1.10, 1.77)	1.17 (1.05, 1.32)	0.006
Model 2	1	1.29 (1.01, 1.65)	1.43 (1.10, 1.85)	1.19 (1.05, 1.35)	0.008
Model 3	1	1.01 (0.78, 1.29)	1.35 (1.04, 1.75)	1.17 (1.02, 1.34)	0.021
Model 4	1	1.02 (0.80, 1.32)	1.37 (1.06, 1.78)	1.18 (1.03, 1.35)	0.014
Dairy protein					
Cases/subcohort	167/239	155/240	154/239	—	
Median (g/d)	10.8	16.8	23.8	—	
Model 1	1	0.91 (0.72, 1.14)	1.07 (0.85, 1.35)	1.03 (0.92, 1.16)	0.56
Model 2	1	0.92 (0.73, 1.16)	1.25 (0.98, 1.58)	1.11 (0.98, 1.25)	0.10
Model 3	1	0.87 (0.69, 1.09)	1.07 (0.84, 1.37)	1.03 (0.91, 1.17)	0.66
Vegetable protein					
Cases/subcohort	158/239	158/240	160/239	—	
Median (g/d)	3.6	5.5	8.7	—	
Model 1	1	1.05 (0.84, 1.32)	1.17 (0.93, 1.46)	1.08 (0.96, 1.21)	0.18
Model 2	1	1.07 (0.85, 1.34)	1.33 (1.05, 1.67)	1.15 (1.02, 1.29)	0.018
Model 3	1	0.97 (0.77, 1.23)	1.04 (0.82, 1.32)	1.02 (0.91, 1.15)	0.75
Cereal protein					
Cases/subcohort	167/239	148/240	161/239	—	
Median (g/d)	4.5	8.4	14.4	—	
Model 1	1	0.78 (0.62, 0.99)	0.78 (0.62, 0.97)	0.88 (0.79, 0.99)	0.030
Model 2	1	0.87 (0.69, 1.10)	0.95 (0.74, 1.21)	0.97 (0.86, 1.10)	0.67
Model 3	1	0.94 (0.74, 1.19)	0.97 (0.75, 1.25)	0.98 (0.86, 1.12)	0.80

1 HRs (95% CIs) and *P*-trend values across tertiles of δ^13^C and δ^15^N and dietary protein types were estimated by Prentice-weighted Cox regression, adjusted for the following: model 1 [sex, family history of diabetes, smoking, education level, physical activity (results for δ^13^C values and δ^15^N values mutually adjusted for each other)], model 2 (model 1 + total energy intake, alcohol intake, and plasma vitamin C), model 3 (model 2 + BMI and waist circumference), and model 4 (model 3 + terrestrial animal protein for δ^13^C, δ^15^N, and fish protein and fish protein for meat protein and terrestrial animal protein). EPIC, European Prospective Investigation into Cancer and Nutrition; δ^13^C, carbon stable-isotope ratio (^13^C/^12^C ratio); δ^15^N, nitrogen stable-isotope ratio (^15^N/^14^N ratio).

2 Total cases: *n* = 476 and subcohort = 718 (includes 16 cases). Median values of isotope and protein tertiles are based on the subcohort only.

3 Terrestrial animal protein is the sum of meat, dairy, and egg protein.

## DISCUSSION

We have described the distribution and correlates of serum carbon (δ^13^C) and nitrogen (δ^15^N) stable-isotope values as potential nutritional biomarkers in a European population-based study and have generated novel findings by examining the association of these measures with incident T2D.

### The distribution of carbon and nitrogen isotope values in a European population

The distribution of serum δ^13^C and δ^15^N in the current study (mean values of −22.8‰ and 10.2‰, respectively) is broadly similar to that reported from small European studies using hair samples (17, 23, 35), with differences in isotopic values being generally attributable to the sample type analyzed. Our (EPIC-Norfolk) population had a relatively low mean (±SD) δ^13^C value (−22.8 ± 0.4‰) compared with North American populations: Atherosclerosis Risk In Communities study (−19.34‰; range: −17.21 to −21.71) and Center for Alaska Native Health Research study (∼−19.8 ± 0.6‰) (21, 22), which was likely attributable to the abundance of C_4_ plants in the food chain in North America (21, 36–38). Conversely, we observed higher mean (±SD) nitrogen isotope values (10.2 ± 0.4‰) than in the CANHR population (8.8 ± 1.4‰) (19), which, given the high intake of marine foods in the CANHR population's diet, was most likely a result of differences in baseline isotope values between the food webs of these populations (39, 40).

### Carbon and nitrogen stable isotope values as nutritional biomarkers

Both δ^13^C and δ^15^N isotope values were most strongly positively associated with dietary fish and fish protein intakes, consistent also with the positive correlation we found with the plasma phospholipid omega-3 fatty acids EPA and DHA. Our observed correlations (ranging between 0.20 and 0.30) are modest in comparison with those observed between EPA, DHA, and erythrocyte δ^15^N (*r* ∼ 0.75) in the CANHR study (19). This was likely explained by the much higher marine-based diet of Yup'ik Eskimo populations than of a general UK population, which typically has a modest fish intake. Overall, our correlations between both δ^13^C and δ^15^N and fish/fish protein in a general population are notably similar in magnitude to those of other biomarkers such as plasma vitamin C as a biomarker of fruit/vegetable intake (41, 42). Our current findings in an observational study of blood-based isotope values also confirm what we previously reported in a small feeding study, ie, higher δ^13^C and δ^15^N were found in urine and fecal samples from individuals after a fish diet when compared with all other diets (18).

Our finding that fish protein intake was associated with δ^13^C and δ^15^N values, which suggests that these are likely biomarkers of fish protein intake, is supported by the elevation of the marine food chain in both δ^13^C and δ^15^N relative to C_3_ terrestrial foods (43, 44). Whereas fish protein in this study only contributed a mean of ∼8% of total dietary protein, consumption varied from 0% to 22%, which allowed for sufficient variation in using it in our analyses. The stronger correlations observed for fish protein than for other types of protein may have been attributable to fish having a very different isotopic signature than other types of protein, with studies of food δ^13^C and δ^15^N values in Europe and Alaska clearly showing much higher δ^13^C and δ^15^N values in marine foods than in nonmarine foods (39, 40).

Whereas δ^13^C values were not associated with animal-source protein intake, δ^15^N values were modestly positively associated with dairy product intake and with lower vegetarian consumer status and with dairy protein, meat protein, and overall terrestrial animal protein intakes—consistent with previous reports that δ^15^N values are markers of primary and secondary animal protein intakes (17, 23, 35). As in the ARIC study (21), we found no significant correlations between δ^13^C and δ^15^N and dietary sugars, except for a modest association of δ^15^N with lactose, which likely reflects that dairy products are higher in δ^15^N than are the sources of other sugars. In contrast with erythrocyte δ^13^C and δ^15^N as potential markers of sweetener intake in the CANHR population (22), and serum δ^13^C as a biomarker of SSB intake in ARIC (21), we did not find an association between SSBs (juice and fizzy drinks) and isotope values. This was most likely explained by the difference in predominant sugar source, which in the United Kingdom, unlike in the United States, is C_3_-derived because the European Union limits the production of high-fructose corn syrup to ∼5% of total sugar production (45, 46). The correlation between alcohol intake and δ^13^C (*r* = 0.20, *P* < 0.001) in our study was unexpected and, to our knowledge, has not been reported previously. This requires further research to elucidate potential mechanisms, because most alcohol consumed in Europe is C_3_-derived (47, 48). A possibility is that alcohol intake may be a marker of other foods rich in δ^13^C (49, 50).

### Association with diabetes

There are several possible explanations for our novel findings of an inverse association of δ^13^C but a positive association of δ^15^N with incident diabetes. It is possible that these opposing associations are driven through the differential dietary intakes reflected by the 2 isotopic values. We noted that only δ^15^N, and not δ^13^C, was a biomarker of terrestrial animal intake and animal protein intake (meat, dairy, and overall terrestrial animal protein intake). A compelling body of evidence exists showing that meat intake, particularly red and processed meat intake, is strongly associated with an increased risk of diabetes (3–6), and it is possible that the positive association of δ^15^N with diabetes in our study largely reflected δ^15^N as a biomarker of meat-related dietary factors. This was supported by our observed positive association of both meat protein intake and terrestrial animal protein intake with diabetes. In contrast, the inverse association of δ^13^C with diabetes was supported by the inverse association of fish protein intake with diabetes in our study and was in line with our previous observation of an inverse association between fish intake and diabetes (10) and with δ^13^C predominantly reflecting its status as a biomarker of fish-related dietary factors. However, there are also issues of measurement error in self-reported intake, which further highlights the need to identify objective nutritional biomarkers. Past research on the association between fish intake and diabetes risk has been inconclusive, showing null associations (8, 9), decreased risk (10), increased risk (51, 52), or variation by geographic location (7, 8), and there has been acknowledgment of the influence of fish-cooking methods and factors other than fish protein, such as omega-3 fatty acid and vitamin D contents that might affect the risk of diabetes. It is also possible that δ^15^N provides a measure of other unknown factors, eg, toxins within fish (53, 54). Hence, toxins may be a correlate of δ^15^N, which could contribute to the positive δ^15^N-diabetes association. Further work is needed to clarify to what extent serum δ^13^C and δ^15^N differentially reflect fish protein intake and whether serum δ^15^N could be used as a biomarker to distinguish the metabolic availability of primary and secondary animal protein (fish, meat, and dairy) in the diet and their isotopic signatures in human blood and tissues.

### Strengths and limitations

This was the largest study to date that examined the distribution and correlates of both δ^13^C and δ^15^N values in a representative European population. It was also the first study that examined the potential use of δ^13^C and δ^15^N values in relation to disease risk. Our findings may be generalizable to other European populations with similar intakes of fish and meat. The limitations of this study merit consideration. Measurement error in FFQ-derived dietary intake may have contributed to the modest correlations with stable isotope values. However, in a subset we examined the association between δ^13^C and δ^15^N and objectively measured plasma phospholipid omega-3 fatty acids, which have been found to be valid biomarkers of fish intake (55). We observed modest correlations between δ^15^N and EPA and DHA of a magnitude similar to that of serum δ^15^N and FFQ-derived fish protein. Measurement error in stable-isotope measures cannot be excluded. We measured stable isotopes in serum, which does not reflect long-term dietary intake, as do commonly used tissues such as hair and nails. However, serum samples were taken at the time the FFQ was administered; therefore, it is unlikely that differences in time between these measures would have had a substantial effect on our findings. Any influence of using nonfasted serum samples on isotope values is unknown, but, if present at all, is likely to be small. For analyses of associations with diabetes, we accounted for a range of confounders, including age and smoking status, which were related to isotopic values, but we cannot exclude the possibility that unmeasured or unknown confounding factors may have influenced our findings. A priori we set out to study associations with diabetes, but it would be of interest to examine the associations of these biomarkers with other chronic disease endpoints in future work.

### Conclusion

We showed in a European population that carbon and nitrogen stable isotope values are potential biomarkers of fish and meat-related intakes that can be measured from stored blood samples in large population-based studies, extending previous findings from small studies of hair samples, from feeding studies, and from specialist populations. We provided the first available evidence that these isotope ratios have potential utility as nutritional biomarkers for investigating disease etiology in relation to diabetes incidence. The divergent diabetes associations with increasing values of δ^13^C (inverse) and δ^15^N (positive) need to be interpreted in light of knowledge of dietary intake and may assist in identifying dietary components that are associated with health risks and benefits. Future research is warranted to help increase our understanding of associations of isotopic signatures with dietary patterns and to establish these biomarkers for dietary assessment in nutritional epidemiology.

## Supplementary Material

Supplemental data

## Footnotes

4 Abbreviations used: ALA, α-linolenic acid; EPIC, European Prospective Investigation into Cancer and Nutrition; FFQ, food-frequency questionnaire; SSB, sugar-sweetened beverage; T2D, type 2 diabetes; δ^13^C, carbon stable-isotope ratio (^13^C/^12^C ratio); δ^15^N, nitrogen stable-isotope ratio (^15^N/^14^N ratio).
