# Supplementary material for: Serum carbon and nitrogen stable isotopes as potential biomarkers of dietary intake and their relation with incident type 2 diabetes: the EPIC-Norfolk study1
Source: Am J Clin Nutr. 2014 Jul 2;100(2):708–18. doi: 10.3945/ajcn.113.068577 (PMC4095667; doi:10.3945/ajcn.113.068577)
Supplement: Supplemental data [file supp_100_2_708__index.html]

Serum carbon and nitrogen stable isotopes as potential biomarkers of dietary intake and their relation with incident type 2 diabetes: the EPIC-Norfolk study — Supplemental data 

# Serum carbon and nitrogen stable isotopes as potential biomarkers of dietary intake and their relation with incident type 2 diabetes: the EPIC-Norfolk study

## Supplemental data

**Files in this Data Supplement:**

- Supplemental data - Tables 1 and 2
