# Supplementary material for: Serum carbon and nitrogen stable isotopes as potential biomarkers of dietary intake and their relation with incident type 2 diabetes: the EPIC-Norfolk study1
Source: Am J Clin Nutr. 2014 Jul 2;100(2):708–18. doi: 10.3945/ajcn.113.068577 (PMC4095667; doi:10.3945/ajcn.113.068577)
Supplement: Supplemental data [file 113.068577_ajcn068577SupplementaryData1.doc]

**Supplementary Table 1:** Distribution of δ^15^N and δ^13^C values by fish and terrestrial animal consumer status in the sub-cohort: The EPIC-Norfolk Study

|  |  | **Mean ± SD** | | |
| --- | --- | --- | --- | --- |
|  |  | **n** | **δ^13^C** | **δ^15^N** |
|  |  |  |  |  |
| Fish consumer groups^†^ (n=369)  (excludes high meat consumers ≥100g/d) | | | | |
| Non-consumer |  | 43 | -22.9 ± 0.4 | 9.9 ± 0.6 |
| Low consumer (0-1 portion/week) |  | 27 | -22.8 ± 0.4 | 10.1 ± 0.3 |
| Medium consumer (1-2 portions/week) |  | 128 | -22.8 ± 0.4 | 10.2 ± 0.4 |
| High consumer (2-3 portions/week) |  | 79 | -22.8 ± 0.3 | 10.4 ± 0.4 |
| Very high consumer (>3 portions/week) |  | 92 | -22.7 ± 0.4 | 10.3 ± 0.4 |
| *P_trend_* |  |  | 0.007 | <0.001 |
|  |  |  |  |  |
| Terrestrial animal & animal products consumer groups (n=296)  (excludes high fish consumers ≥200g/week)^‡^ | | | | |
| Low consumer (<250g/d) |  | 28 | -23.0 ± 0.4 | 9.8 ± 0.6 |
| Medium consumer (250-<500g/d) |  | 118 | -22.8 ± 0.4 | 10.2 ± 0.4 |
| High consumer (500-<750g/d) |  | 118 | -22.9 ± 0.3 | 10.2 ± 0.4 |
| Very high consumer (>750g/d) |  | 32 | -22.9 ± 0.3 | 10.2 ± 0.3 |
| *P_trend_* |  |  | 0.690 | 0.001 |
|  |  |  |  |  |
| Values are means (SD), with P for trend analyses corresponding to linear regression analyses.  ^†^Range of portions/week in fish consumer groups: portions are > for lower end of interval and ≤ for the upper interval.  ^‡^Terrestrial animal and animal products consumer group includes those that consume meat, dairy, eggs. | | | | |

**Supplementary Table 2:** Baseline characteristics of the study population by incident diabetes case and sub-cohort status (n=1,178): The EPIC-Norfolk study

| **Characteristic** | **Subcohort*** | **Cases** | **P-value** |
| --- | --- | --- | --- |
| **n** | 718 | 460 | - |
| ***Socio-demographic characteristics*** |  |  |  |
| Age (yrs)^1^ | 57.7 (9.3) | 60.8 (8.3) | <0.001 |
| Sex – men n(%)^2^ | 294 (41.0) | 251 (54.6) | <0.001 |
| BMI (kg/m^2^)^1^ | 25.9 (3.6) | 29.5 (4.4) | <0.001 |
| Waist circumference (cm)-Men^1^ | 94.5 (9.6) | 103.0 (9.7) | <0.001 |
| Waist circumference (cm)-Women^1^ | 80.7 (9.9) | 92.4 (12.2) | <0.001 |
| Family history of diabetes – *yes* n(%)^2^ | 79 (11.0) | 118 (25.7) | <0.001 |
|  |  |  |  |
| Smoking status^2^ |  |  | 0.001 |
| Never | 348 (48.5) | 192 (41.7) |  |
| Former | 275 (38.3) | 224 (48.7) |  |
| Current | 95 (13.2) | 44 (9.6) |  |
|  |  |  |  |
| Education level n(%)^2^ |  |  | <0.01 |
| School compulsory education | 264 (36.8) | 205 (44.6) |  |
| Up to O level | 70 (9.8) | 44 (9.6) |  |
| Up to A level | 284 (39.6) | 175 (38.0) |  |
| Up to degree level | 100 (13.9) | 36 (7.8) |  |
|  |  |  |  |
| Physical activity n(%)^2^ |  |  | <0.001 |
| Active | 15 1 (21.0) | 81 (17.6) |  |
| Moderately active | 187 (26.0) | 93 (20.2) |  |
| Moderately inactive | 202 (28.1) | 102 (22.2) |  |
| Inactive | 178 (24.8) | 184 (40.0) |  |
| ***Dietary characteristics*** |  |  |  |
|  |  |  |  |
| Total energy intake (kcal/d)^1^ | 2047 (604) | 2055 (621) | 0.81 |
| Alcohol intake (g/d)^3^ | 3.9 (0.8, 10.3) | 2.7 (0, 10.5) | 0.07 |
| Fruit intake (g/d)^3^ | 212.7 (124.7, 338.2) | 193.6 (97.9, 317.0) | 0.01 |
| Vegetable intake (g/d)^3^ | 245.5 (186.6, 334.7) | 249.6 (174.5, 334.2) | 0.42 |
| Plasma vitamin C (μmol/l)^3^ | 55 (42, 66) | 44 (32, 57) | <0.001 |
| Fish intake (g/d)^3^ | 32.1 (22.4, 46.6) | 32.1 (19.3, 47.6) | 0.37 |
| Meat intake (g/d)^3^ | 98.1 (65.6, 128.3) | 106.6 (77.1, 141.6) | <0.001 |
| Total breakfast cereals (g/d) | 12.9 (2.1, 30.0) | 12.9 (2.1, 30.0) | 0.47 |
| Fizzy drink (g/d) | 0.0 (0.0, 28.0) | 0.0 (0.0, 28.0) | 0.47 |
| Fruit juice (g/d) | 16.8 (0.0, 94.8) | 8.4 (0.0, 51.6) | 0.04 |
|  |  |  |  |
| Follows a vegetarian diet – *yes* n(%)^2^ | 37 (5.2) | 20 (4.4) | 0.53 |
| Fish consumption level^†^ n(%)^2^ |  |  | 0.23 |
| Non-consumer | 43 (11.7) | 26 (12.8) |  |
| Low consumer | 27 (7.3) | 16 (7.9) |  |
| Medium consumer | 128 (34.7) | 58 (28.6) |  |
| High consumer | 79 (21.4) | 36 (17.7) |  |
| Very high consumer | 92 (24.9) | 67 (33.0) |  |
|  |  |  |  |
| Terrestrial animal consumption level^†^ n(%)^2^ |  |  | 0.14 |
| Low consumer | 28 (9.5) | 11 (5.6) |  |
| Medium consumer | 118 (39.9) | 92 (46.7) |  |
| High consumer | 118 (39.9) | 67 (34.0) |  |
| Very high consumer | 32 (10.8) | 27 (13.7) |  |
|  |  |  |  |
| Total protein (g/d)^3^ | 81.6 (66.6, 95.5) | 84.0 (67.6, 97.3) | 0.13 |
| Fish protein (g/d)^3^ | 5.9 (3.6, 8.4) | 5.9 (3.6, 8.6) | 0.28 |
| Meat protein (g/d)^3^ | 23.7 (15.9, 31.2) | 25.2 (18.1, 34.8) | <0.001 |
| Dairy protein^†^ (g/d)^3^ | 16.8 (12.9, 21.5) | 16.8 (12.4, 22.1) | 0.99 |
| Vegetable protein (g/d)^3^ | 5.4 (4.0, 7.4) | 5.7 (3.8, 7.6) | 0.82 |
| Cereal protein (g/d)^3^ | 8.4 (5.6, 12.3) | 8.9 (5.7, 12.5) | 0.65 |
| **Mean serum δ^15^N (‰)**^1^ | 10.2 (0.4) | 10.3 (0.4) | 0.006 |
| Range (min-max) | 8.0 to 11.7 | 8.8 to 11.5 | - |
| **Mean serum δ^13^C (‰)^1^** | -22.8 (0.4) | -22.9 (0.4) | 0.009 |
| Range (min-max) | -23.8 to -21.1 | -24.0 to -21.5 | - |
| *Sub-cohort includes n=16 cases (described in methods). Values are means (SD)^1^, frequencies (percentages)^2^, and medians (inter-quartile range)^3^ and corresponding P-values correspond to t-test, χ2 test, or Kruskal-Wallis test.  ^†^ n=369 (n=13 cases in the sub-cohort) for those following a fish diet and n=296 (n=10 cases in the sub-cohort) for those following a terrestrial meat diet. | | | |
